# Dr. William G. Brownell

**Published:** 1915-05

**Authors:** 


**Dr. William G. Brownell,** N. Y. Homoeopathic 1879, died in Rochester recently, aged 60.

